# Sustainable Rearing of *Tenebrio molitor* Larvae Using Peatland Biomass

**DOI:** 10.3390/insects17040436

**Published:** 2026-04-18

**Authors:** Asma Akaichi, Nazanin Fazel Dehkordi, Jan Berend Lingens, Alexandra Rath, Florian Lohkamp, Amr Abd El-Wahab, Marwa F. E. Ahmed, Nils Th. Grabowski, Kashif ur Rehman, Madeleine Plötz, Christian Visscher, Cornelia Schwennen

**Affiliations:** 1 Institute for Animal Nutrition, University of Veterinary Medicine Hannover, Foundation, 30173 Hannover, Germany; asmaakaichi@gmail.com (A.A.); nazanin.fazel.dehkordi@tiho-hannover.de (N.F.D.); jan.berend.lingens@tiho-hannover.de (J.B.L.); alexandra.rath@tiho-hannover.de (A.R.); florian.lohkamp@tiho-hannover.de (F.L.); amr.abd.el-wahab@tiho-hannover.de (A.A.E.-W.); marwa.ahmed@tiho-hannover.de (M.F.E.A.); christian.visscher@tiho-hannover.de (C.V.); 2 Department of Nutrition and Clinical Nutrition, Faculty of Veterinary Medicine, Mansoura University, Mansoura 35516, Egypt; 3 Department of Hygiene and Zoonoses, Faculty of Veterinary Medicine, Mansoura University, Mansoura 35516, Egypt; 4 Institute of Food Quality and Food Safety, University of Veterinary Medicine Hannover, Foundation, Bischofsholer Damm 15, 30173 Hannover, Germany; nils.grabowski@tiho-hannover.de (N.T.G.); madeleine.ploetz@tiho-hannover.de (M.P.); 5 German Institute of Food Technologies (DIL e.V.), Prof.-v-Klitzing-Str. 7, 49610 Quakenbrueck, Germany; k.rehman@dil-ev.de

**Keywords:** mealworms, biomass recycling, rewetted peatlands, growth performance, nutritional composition

## Abstract

To promote sustainable biomass recycling and support food security, *Tenebrio molitor* (TM) larvae can serve as an eco-friendly source of food and feed. This study compared the survival, growth performance, and nutritional composition of TM larvae fed five diets. The control (CON) diet contained distillers’ dried grains with solubles (DDGS) and wheat bran (WB), while the experimental diets included 10–40% lignocellulose-rich organic products from rewetted peatlands (LPRP) replacing WB, with DDGS adjusted to maintain equivalent protein levels (about 21%). A total of 2500 larvae were divided into five replicates per treatment (100 larvae each). Survival exceeded 90% across all groups. Larvae fed the CON diet had a higher final body weight than those on the 30% and 40% LPRP diets (*p* < 0.05), with no significant differences among the CON and 10% and 20% LPRP groups. The feed conversion ratio (fresh matter) was significantly lower in the CON and 10% LPRP groups than in the other groups (*p* < 0.05). Larvae fed the 10% LPRP diet showed slightly higher crude protein content (55.8%) compared to the control group (54.8%) and the other treatment groups, whereas those fed the 30% LPRP diet had the highest numerical total amino acid content. Taken together, these results indicate that incorporating 10% LPRP with DDGS and WB provides the best overall balance between growth performance and nutritional quality for TM larvae, supporting sustainable production and circular economy goals.

## Simple Summary

Finding sustainable ways to produce food and feed is becoming increasingly important as the global population grows. Mealworms, the larvae of the darkling beetle *Tenebrio molitor*, can turn low-value plant materials into nutritious biomass rich in protein and other valuable nutrients. This study tested whether materials from rewetted peatland restored to increase CO_2_ binding could be used as part of mealworm feed. Five different diets were tested in mealworm feed: one standard diet and four experimental diets in which the wheat bran was partially replaced with 10–40% peatland plant material. All diets had the same protein level (21%). More than 90% of the larvae survived on all diets. Larvae fed the control diet and the diet containing 10% peatland material converted feed into body weight (fresh matter) more efficiently than those fed the diets containing 20%, 30% and 40% peatland material. Larvae fed 10% peatland material also showed slightly higher protein content compared to the other groups. These results suggest that including 10% peatland material in mealworm feed can be a sustainable option. Using such recycled plant materials can help reduce waste, support circular farming systems, and possibly produce environmentally friendly protein sources.

## 1. Introduction

According to the Food and Agriculture Organization of the United Nations, the demand for animal protein is projected to increase by 20% by 2050 compared to 2020 levels [1]. This growing demand places increasing pressure on traditional agriculture and livestock production systems, prompting food scientists and the agri-food industry to explore innovative approaches that reduce environmental impacts and reduce or replace meat consumption with alternative protein sources. Insects have been identified as a promising solution in this search, as they are considered a sustainable source of dietary protein for both humans and animals [2,3], in part having a much lower environmental impact compared to traditional livestock, making them an appealing option for sustainable food and feed systems [4,5]. The use of insects as food and feed offers significant potential due to their rich nutritional profile, including high-quality protein, minerals, and, in some species such as larvae of the saturniid moth *Cirina butyrospermi*, polyunsaturated fatty acids [6].

Yellow mealworm larvae (*Tenebrio molitor*, TM) are among the few insect species approved by legislation for human consumption and are being mass-reared across the European Union (EU) [7,8]. Observing EU and national feed legislation, TM can be reared on various substrates such as flour, grains, and food by-products [9]. TM larvae exhibit functional properties that make them a promising protein source [2]. Their protein content averages around 54.8%, with lysine accounting for approximately 5% of total protein [10]. They are also rich in essential amino acids and unsaturated fatty acids, including omega-3 and omega-6, further enhancing their value as a sustainable protein source [11].

Mealworm rearing typically involves wheat bran and plant-based moisture sources, such as fresh carrots, to ensure adequate nutrition [12]. However, these substrates are also valuable feed ingredients for livestock [13]. Therefore, identifying alternative feeding sources is essential. Recent research has focused on valorizing agricultural and agro-industrial by-products, such as maize stover, carrot tops, orange peel, cabbage stems, spent grains, and bread residues, as substrates for TM larvae [14,15,16]. To date, however, no studies have investigated the use of lignocellulose-rich organic products from rewetted peatlands (LPRP) for mealworm rearing.

Peatlands are among the Earth’s largest natural carbon reservoirs, storing vast amounts of organic carbon that play a crucial role in regulating the global climate [17]. They cover about 4.2 million km^2^ worldwide, mainly across northern latitudes (45–65° N), with major distributions in Asia, North America, and Europe [18]. Formed under waterlogged, low-temperature conditions that slow microbial decomposition, peatlands typically feature peat layers ≥ 30 cm thick and organic matter contents above 30% [19]. Owing to their high carbon density and accumulation rates, they contain the highest carbon stocks per unit area among terrestrial ecosystems [20]. Beyond carbon storage, peatlands contribute to freshwater supply, flood regulation, biodiversity conservation, and support local livelihoods, underscoring their global ecological and socioeconomic importance [17,18]. Despite their ecological importance, many peatlands have been extensively drained for agriculture and forestry, resulting in altered greenhouse gas fluxes and changes in ecosystem functioning [21,22]. In response, peatland rewetting has been proposed as a climate mitigation strategy aimed at reducing CO_2_ emissions and improving the long-term carbon balance of these ecosystems [23]. Rewetting and restoration activities have been shown to alter plant community composition and biomass allocation, with only partial recovery of the natural vegetation structure [24]. This process can promote the accumulation of fast-growing wetland vegetation, resulting in lignocellulose-rich biomass with low direct economic value. Biomass produced from paludiculture on rewetted peatlands is often difficult to utilize economically, particularly for energy applications such as biogas production, which under current conditions is generally unfeasible [25]. Therefore, innovative recycling strategies are required to ensure the sustainable utilization of this biomass.

Among the available options, TM larvae represent an eco-friendly solution due to their ability to convert organic residues into protein-rich biomass [26,27]. As mealworms can thrive on a wide range of agricultural and organic materials [13,28], incorporating LPRP into their diet offers dual benefits: recycling rewetted peatland biomass and producing high-quality insect protein for feed applications.

This study aims to evaluate the feasibility of using LPRP as a feed component for yellow mealworms, emphasizing the interconnected goals of circularity, sustainable agriculture, and ecosystem preservation. By combining LPRP with agro-industrial by-products to formulate balanced diets for TM larvae, this research seeks to explore approaches that could support more sustainable agricultural practices and contribute to peatland restoration and environmental health.

## 2. Materials and Methods

### 2.1. Diet Preparation

Lignocellulose-rich organic products from rewetted peatlands (LPRP) and two industrial by-products, distillers’ dried grains with solubles (DDGS) and wheat bran (WB), were selected for the experiment. The LPRP consisted of biomass derived from rewetted peatland vegetation and was primarily composed of lignocellulosic plant material. The chemical composition of LPRP, including crude protein, crude ash, crude fiber, and crude fat, is presented in Table 1. The material was characterized by a high fiber content, with neutral detergent fiber, acid detergent fiber, and acid detergent lignin values of 717.9, 443.8, and 97.65 g/kg dry matter, respectively, indicating a highly lignocellulosic substrate. The LPRPs were obtained from the project “Klimafarm” (Stiftung Naturschutz Schleswig-Holstein, Erfede, Germany). DDGS and WB were supplied by commercial companies (DDGS: Mischfutter Werke Mannheim GmbH, Mannheim, Germany; WB: Mundt Hans-Wilhelm jun. Landhandel, Lehrte, Germany).

The experimental diets were formulated using three feed materials with distinct chemical compositions: (1) LPRP, (2) DDGS, and (3) WB (Table 1). Five diets, each with a similar protein content (approximately 21%), were tested. The control diet (CON) consisted of DDGS and WB only, while the other four diets contained increasing inclusion levels of LPRP (10%, 20%, 30%, and 40%), replacing wheat bran, while DDGS levels were adjusted to maintain an equivalent protein concentration.

For diet preparation, DDGS and LPRP were ground to achieve a particle size of approximately 3 mm. Wheat bran was not ground, as its particle size was already comparable to that of the other ingredients (approximately 3 mm). The mixtures were then sieved through a 400 µm mesh to remove fine particulate matter, including particles resembling larval frass; preliminary observations indicated that they were predominantly smaller than this threshold. The nutritional composition of all feeds was analyzed for crude ash, crude protein, crude fiber, and crude fat (Table 2). The chemical composition analysis methods are described in Section 2.3. Amino acid profiles were analyzed in individual ingredients and used to calculate dietary amino acid compositions (Table 3).

### 2.2. Insect Rearing, Larval Growth, and Development Measurements

A total of 2500 TM larvae, approximately 35 days old, were used in this study. They were reared at the Institute for Animal Nutrition, University of Veterinary Medicine Hannover, Foundation, Hanover, Germany, under controlled environmental conditions (25–27 °C, 50–60% relative humidity, 12 h photoperiod). The environmental parameters were set according to Van Broekhoven et al. [29]. Newly emerged larvae were pre-fed ad libitum with WB and provided fresh carrots as a water source for five weeks. This regimen promoted development to the 4th–5th-instar stage, making them suitable for collection and uniform allocation to experimental treatments.

At the start of the trial, larvae were randomly distributed into 25 boxes across five dietary treatments (five replicates per treatment). Each replicate consisted of 100 larvae placed in plastic containers (33 × 19.5 × 12 cm). Each box initially received 6 g of experimental feed weekly and 4 g of carrots twice weekly as a moisture source. Feed and carrot quantities were adjusted according to larval intake.

Feed consumption (offered and rejected feed), mortality, and larval weight were recorded weekly. Mean larval weight and mean weight gain per larva were calculated accordingly. Frass was removed weekly by sieving through a 300 µm mesh from week 6, and through 400 µm and 500 µm sieves from week 8, corresponding to the increase in fecal particle size. When the first pupae appeared, all larvae were counted and fasted for 24 h before harvest. Larvae were then weighed and euthanized by freezing at −20 °C.

To determine feed consumption on a dry matter basis, rejected feed and carrots were collected weekly, weighed, and dried at 103 °C for 24 h until a constant mass was achieved. Feed intake (fresh and dry matter) was calculated by subtracting the residual feed from the initial amount offered. The same method was applied for carrots, which were assessed twice weekly.

Based on these data, the feed conversion ratio (FCR) was calculated. FCR was determined by dividing the total mean individual feed intake by the total mean individual weight gain, expressed on both fresh and dry matter bases for feed and carrots, while larval weight gain was expressed on a fresh matter basis [30,31].

### 2.3. Chemical Composition Analysis

The TM larvae, substrates, and feeds were analyzed for their nutritional composition using the procedures from the Association of German Agricultural Analytical and Research Institutes (VDLUFA) [32] at the Institute for Animal Nutrition, University of Veterinary Medicine Hannover, Foundation. The dry matter content was calculated by weighing the samples before and after drying them at 103 °C for 24 h. The muffle furnace was used to determine the crude ash content of the dried and ground samples by combustion at 600 °C for 6 h. The total N content was also measured using the Dumas incineration method (rapid MAX N exceed, Elementar Analysensysteme GmbH, Langenfeld, Germany). Then, the crude protein content was calculated by multiplying the nitrogen content by a factor of 6.25 (also for larva samples as shown by Oonincx et al. [30]; Kröncke and Benning [33]). The crude fiber content was determined by washing samples in diluted acidic and alkaline solutions, followed by drying at 103 °C (FIBRETHERM FT 12, C. Gerhardt GmbH & Co. KG, Königswinter, Germany). The samples were then ashed for 5 h at 520 °C in a muffle furnace. Crude fat was hydrolyzed with hydrochloric acid (w = 15%) (Hydrotherm V02, C. Gerhardt GmbH & Co. KG, Königswinter, Germany), and extracted with petroleum ether with a Soxhlet apparatus (Soxtherm 416, C. Gerhardt GmbH & Co. KG, Königswinter, Germany). The flask was dried for 90 min at 103 °C and reweighed after cooling in a desiccator. Finally, the amino acid content, excluding tryptophan (which was not analyzed), was determined using Biochrom 30+ (Laborservice Onken GmbH, Gründau-Breitenborn, Germany).

### 2.4. Statistical Analysis

Statistical analyses were performed using the Statistical Analysis System for Windows, SAS^®^ version 7.1 (SAS Institute Inc., Cary, NC, USA). Growth performance parameters were analyzed for each replicate (*n* = 5 per treatment). Mean values and their corresponding standard deviations were calculated. The normality of all data was evaluated using the Shapiro–Wilk test.

When data followed a normal distribution, differences among treatments were tested using the Ryan–Einot–Gabriel–Welsch test. For non-normally distributed data, the Kruskal–Wallis test was applied, followed by the Wilcoxon two-sample test to assess pairwise differences. Differences were considered statistically significant at *p* < 0.05.

Statistical analysis of the chemical composition of larvae was not performed due to the insufficient number of larvae per replicate. Instead, samples from all five replicates were pooled into two composite samples (*n* = 2 per treatment) to obtain the minimum required quantity for chemical analysis. This pooling approach allowed for detectable measurements but did not permit replicate-level statistical evaluation.

## 3. Results

### 3.1. Growth Performance

**Figure 1:**
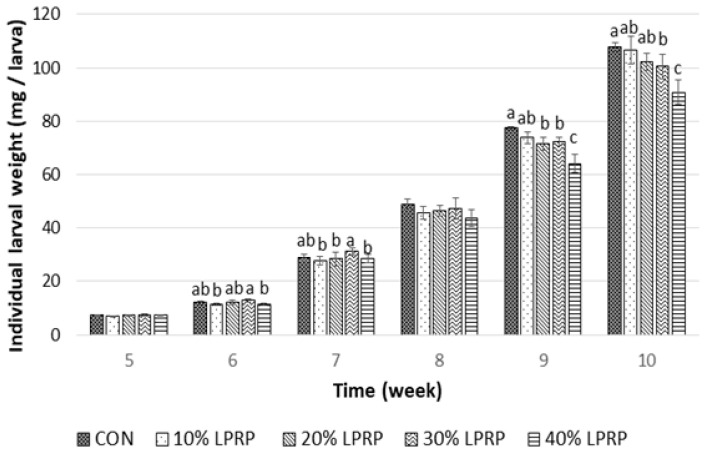
Individual larval weight depends on the type of diet and week of rearing; CON: Control diet; LPRP (lignocellulose-rich organic products from rewetted peatlands). ^a,b,c^ Different letters indicate statistical difference (*p* < 0.05) among the larval weight fed with different diets at the same rearing time. Values are expressed as mean ± standard deviation (*n* = 5 replications).

Individual larval weights differed significantly among the treatment groups (*p* < 0.05) (Figure 1). During the first two weeks of the experiment, the highest weight was observed in larvae fed a diet containing 30% LPRP, with a significant difference between this group and the 10% and 40% LPRP groups (*p* < 0.05). However, in weeks 9 and 10 (final two weeks of the experiment), mealworms fed the CON diet reached the highest body weight compared to those fed the 30% and 40% LPRP diets. At the final measurement (week 10), the highest and lowest individual larval weights were recorded for mealworms grown on the CON diet (108 mg ± 1.73) and the 40% LPRP diet (90.8 mg ± 4.50), respectively (*p* < 0.05). TM larvae fed the CON diet exhibited a higher final body weight compared to those fed the 30% and 40% LPRP diets (*p* < 0.05), while no significant differences were observed compared to the 10% and 20% LPRP diets. The final body weight of larvae fed the 30% LPRP diet was significantly higher than those fed the 40% LPRP diet, but lower than those fed the CON diet (*p* < 0.05).

**Figure 2:**
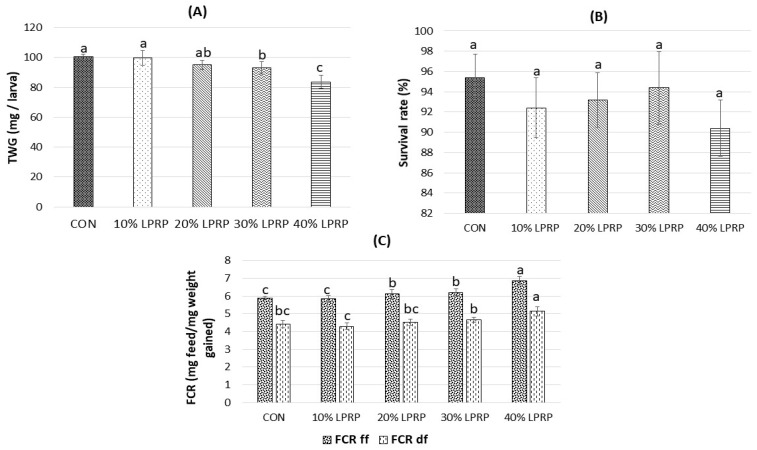
Growth performance of *T. molitor* after rearing with different diets; (**A**) total weight gain (TWG); (**B**) survival rate (%); (**C**) feed conversion rate (FCR) based on fresh and dry basis. CON: Control diet; LPRP (lignocellulose-rich organic products from rewetted peatlands). ^a,b,c^ Different letters indicate statistical difference (*p* < 0.05) among the larvae fed with different diets. Values are expressed as mean ± standard deviation (*n* = 5 replications).

The results for the productive performance and survival rate of the yellow mealworms are shown in Figure 2. The total weight gain (Figure 2A) was affected by the level of inclusion of LPRP in the diet (*p* < 0.05). The highest total weight gain was observed in mealworms fed the CON diet, closely followed by those fed the 10% LPRP diet, which was higher than that observed in the 30% and 40% LPRP groups. In contrast, inclusion of 40% LPRP resulted in the lowest individual larval total weight gain compared to the other groups (*p* < 0.05). In addition, similar total weight gains for one larva were found for the CON and 10% and 20% LPRP groups (100 mg, 99.8 mg, and 95.1 mg for CON, 10% LPRP, and 20% LPRP, respectively), and these were significantly higher than those for the 40% LPRP group (83.6 mg) (*p* < 0.05). For all five groups, the diets did not significantly affect the survival rate (Figure 2B). The survival rates ranged between 90.4% and 95.4% for larvae fed the 40% LPRP diet and the CON diet, respectively.

The type of diet influenced the FCR based on the fresh and dry matter (*p* < 0.05), with statistical differences between the 40% LPRP diet and the other diets (Figure 2C). The lowest FCR based on fresh feed was observed in the CON and 10% LPRP groups compared with the other treatments (*p* < 0.05). In contrast, the 40% LPRP diet showed the highest FCR based on dry matter compared with the other groups (*p* < 0.05), whereas no significant differences were found among the CON and 10% and 20% LPRP groups. The 10% LPRP group showed a more favorable FCR based on dry matter than the 30% and 40% LPRP groups (*p* < 0.05).

### 3.2. Larval Chemical Composition

The contents of dry matter, crude ash, crude protein, and crude fat of larvae are shown in Table 4. The dry matter content was numerically highest in mealworms reared on the CON diet, while the crude ash content was numerically highest in larvae reared on the 40% LPRP diet. The 10% LPRP diet resulted in a numerical increase in the crude protein content of mealworms compared to the other diets, whereas mealworms reared on the 40% LPRP diet had the numerically lowest crude protein content. Additionally, the crude fat content was numerically higher in mealworms reared on the 40% LPRP diet.

Table 5 shows the amino acid composition of the TM larvae according to their rearing diet. Alanine, aspartic acid, and glutamic acid are among the more abundant amino acids, while methionine and cysteine are less prevalent in the proteins of all groups. Arginine, leucine, lysine, and valine are the most abundant essential amino acids. In contrast, alanine, aspartic acid, glutamic acid, proline, and tyrosine are the most common non-essential amino acids in TM larvae across all groups. TM larvae fed a 30% LPRP diet had the numerically highest total amino acids, whereas those on a 10% LPRP diet had the numerically lowest total amino acids and total essential amino acids (Table 5).

## 4. Discussion

### 4.1. Growth Performance and Survival Rate

In terms of performance, larval weight is a critical indicator in insect rearing. The increase in the weight of larvae grown on a 30% LPRP diet during the second week of the experimental period, followed by a decrease in weight gain for the remaining period, was similar to the trend noted by Li et al. [34]. They observed that as the crude fiber content of the feed rose (from 0.37% to 20.4% crude fiber), larval growth initially increased but then declined. In another study, a higher final fresh weight (about 200 mg/larva) was observed compared to our CON group, where mealworms were fed for 16 weeks with a mixture of WB and DDGS, and provided with cabbage leaves as a water source, whereas carrots were used as the water source in our study [35]. The composition of the feed is an important factor for the growth of insects. The nutritional analysis of the CON and the experimental diets showed similar crude protein levels (about 21%); however, diets with higher LPRP inclusion contained progressively fewer total amino acids. This is reflected in the decreasing ratio of summed amino acids to crude protein, which dropped from 97.1% in the control diet to 86.2% at 40% LPRP inclusion, indicating an increasing contribution of non-protein nitrogen. As non-protein nitrogen does not supply essential amino acids, this reduction in amino acid availability may have contributed to the observed constraints on larval growth and performance at higher LPRP inclusion levels.

Similarly, the carbohydrate and crude fat contents showed differences of no more than 2.2 percentage points, suggesting a negligible influence on larval growth. In contrast, the crude fiber content emerged as a key factor influencing TM larval performance. An increase in the crude fiber content decreased the weight gain of mealworms. The diet containing 40% LPRP likely had an inadequate nutritional composition for mealworms due to it having the highest fiber content (19.1% dry matter) compared to the other diets. A similar outcome was observed in a study by Li et al. [34] that noted that the highest and lowest weights were recorded in larvae fed diets with 5% and 20% crude fiber levels, respectively. That study indicated that a crude fiber content of approximately 20% could be excessive for the optimal growth of TM larvae, potentially leading to malnutrition stress. Similar findings were reported by Montalbán et al. [10] and Morales-Ramos et al. [10,36], indicating that the incorporation of byproducts with high fiber content in the diet negatively affects the development of the larvae.

The literature reports substantial variability in FCR, which is largely affected by the dietary composition offered [29,30,37]. In this study, the fiber-rich diet (40% LPRP) showed a higher FCR compared to the other diets. According to Montalbán et al. [13], diets with high fiber content, as indicated by elevated Neutral Detergent Fiber (NDF) and Acid Detergent Fiber levels, lead to decreased digestibility. Moreover, a diet comprising cellulose can lead to the colonization of the gut microbiota by anaerobic bacteria like *Parabacteroides* spp. and *Clostridium* spp., which play a key role in breaking down hemicellulose in natural environments [38]. In fact, mealworms possess basic needs for their digestive systems to function properly, with a crude fiber level between 5% and 10% being necessary for the larvae to achieve optimal growth and development [34]. In the present study, the fiber content of all diets was higher than 10%, which could explain the worsening in the FCR in all the experimental diets we administered. Ruschioni et al. [39] observed a negative effect on TM larvae when the crude fiber content exceeded 16.8%. In the current study, the FCR calculated on a fresh weight basis was notably higher than that reported in previous studies, which used WB as a control diet [28,40]. However, it aligns with findings from another study [30] that examined a diet with slightly higher protein content (22.9% dry matter) but lower fat content compared to our experimental diets, which also included carrots as a moisture source to support mealworm growth. That same study reported that when FCR calculations were based solely on concentrated feed and excluded carrots, the FCR values for carrot-supplemented diets decreased from 4.5 to 1.8 for the high-protein, high-fat diet. This finding (FCR value decrease) suggests that the method of providing water in the diet is crucial for the performance of TM, as previously observed [28,29,30,37]. The use of carrots as a moisture source can influence FCR [37]. However, given the small difference in carrot consumption in the current study, the observed differences in FCR are unlikely to be attributable to carrots. Therefore, the high FCR in the 40% LPRP diet is more likely due to the lower digestibility and poorer nutritional quality of the LPRP-containing diet.

The survival rate was over 90% and did not significantly differ among the experimental groups, with values better than those reported by other authors [28,30]. This finding implies that all diets, formulated with the recommended protein ratio for TM larval growth, were accepted by mealworms [41]. Therefore, all diets were adequate for larval growth and development.

### 4.2. Larval Chemical Composition

The level of inclusion of LPRP in the diet influenced the chemical composition of mealworms. The dry matter contents of TM larvae grown on the CON and 10% and 20% LPRP diets presented here were higher than those reported in other studies [30,37], despite the diets having a similar crude protein content of around 21%. The crude ash content of mealworms from all groups was comparable to that reported by López-Gámez et al. [28], who found that the crude ash content of mealworms grown on WB supplemented with vegetable residues ranged between 3.94% and 4.15%. Interestingly, the larvae reared on the 40% LPRP diet, which had the lowest ash content among all diets, showed the highest crude ash content in their bodies. This pattern was accompanied by a slightly higher carrot intake in this group compared to the control. As carrots contain carbohydrates and minerals such as calcium, iron, and magnesium [42], this intake may have marginally contributed to larval mineral content; however, given the small difference in carrot consumption, this contribution is likely limited. Other physiological or metabolic factors may therefore also have played a role.

There has been a controversial debate over the impact of diet on the protein content of larvae. Montalbán et al. [13] reported that increasing the crude protein content in the diet correlates with an increase in the protein content of TM larvae. Mancini et al. [43] found that by using cereal byproduct-based diets, the dietary protein content increased and the protein content of the larvae also increased. In our experiment, we observed some deviations from the expected trend. Although crude protein content in the feed was nearly identical across all diets, larval protein levels varied among dietary treatments, indicating that protein deposition is influenced not only by dietary protein concentration but also by factors such as protein digestibility and amino acid availability. Diets with higher proportions of LPRP may have reduced protein digestibility, possibly because some amino acids in the LPRP material are bound to fiber (NDF) and are therefore less accessible for absorption. In an experimental study, broilers fed diets with added lignocellulose (a high-fiber component) showed impaired nutrient digestibility, including protein, when fiber content increased, suggesting that fiber can limit overall protein and amino acid availability [44]. This would result in fewer digestible amino acids and lower incorporation into larval biomass. A similar finding was reported in a study by Bordiean et al. [37], where the protein content of larvae increased on diets with comparable crude protein levels varying significantly, with values of 50.9% and 53.4%, respectively.

In the present study, crude protein was calculated from total nitrogen using the universal nitrogen-to-protein conversion factor of 6.25. However, as reported in previous studies on insects, this approach may lead to an overestimation of protein content due to the presence of non-protein nitrogen compounds such as chitin and nucleic acids [45,46]. This was supported by expressing the sum of amino acids relative to the calculated crude protein, which accounted for approximately 89.3–99.6% of the crude protein content. These values indicate that the majority of the nitrogen measured by the Dumas incineration method was incorporated into true protein, while the remaining fraction likely reflects non-protein nitrogen and methodological limitations of the universal conversion factor. Similar discrepancies between crude protein and amino acid-based assessments have been reported in the literature, where protein values calculated using the 6.25 factor were higher than those inferred from amino acid data [46,47]. Ritvanen et al. [47] demonstrated that applying a lower, species-specific nitrogen-to-protein conversion factor resulted in protein values more closely aligned with the amino acid composition [47]. Therefore, the difference observed in the present study between crude protein and the summed amino acids likely reflects a modest overestimation of protein content associated with the use of the universal conversion factor.

The levels of alanine, aspartic acid, and glutamic acid were the highest, while the levels of methionine and cysteine were the lowest in all groups. The same observation was noted by Yu et al. [40]. Among the essential amino acids, arginine, leucine, lysine, and valine were present at the highest levels. These amino acids are considered nutritionally important due to their roles in muscle development, immune function, energy metabolism, and stress response in animals and livestock, thereby contributing to the nutritional quality of mealworm larvae as a feed or food source [48,49]. The presence of high levels of essential amino acids enhances the nutritional quality and functional value of mealworm larvae as a protein ingredient. In particular, amino acid-rich insect biomass can partially replace conventional protein sources such as soybean meal, as TM larvae contain relatively high levels of lysine, methionine, threonine, tryptophan, valine, and isoleucine compared with soybean meal [50]. This may also reduce the need for synthetic amino acid supplementation in feed formulations. These characteristics improve the economic attractiveness of insect production, as TM exhibits a comparatively richer and more balanced essential amino acid profile than black soldier fly larvae, particularly due to higher levels of certain amino acids such as methionine and a more diverse overall amino acid distribution [51,52]. Consequently, mealworm meals with well-balanced essential amino acid profiles may achieve higher value in both feed and food markets.

Glutamic acid, a crucial component of nitrogen assimilation [53], was the most abundant non-essential amino acid found in the larvae of all groups. Alanine, acid aspartic, glycine, proline, and tyrosine are the most common non-essential amino acids in TM larvae [40]. The results of the current study suggest that the amino acid composition of larvae is not determined solely by the crude protein content of the diet. Although the diets had similar protein levels, differences in larval amino acid profiles were observed, which may reflect variations in the amino acid composition of the feed ingredients, as these were not fully standardized across diets. Additionally, because larvae must obtain essential amino acids from their diet, differences in the availability of these amino acids could contribute to the observed variation [54].

The total fat content differed among larvae fed with different experimental diets. Larvae cultivated on the substrate containing 40% LPRP exhibited the highest fat content. In general, the crude fat content of larvae grown on different proportions of LPRP in the present study was higher than that reported in other studies [28,29,30,37], making LPRP a suitable substrate for obtaining a valuable source of dietary fat. This finding aligns with that of Montalbán et al. [10], who reported that diets with higher fiber content led to increased fat content and higher FCR. Insects can synthesize lipids from different dietary components, such as carbohydrates [55], which might explain the increased total lipid content in those larvae supplemented with 40% LPRP. Tsuchida and Wells [56] reported that a significant portion of an insect’s body fat consists of triglycerides, which can be synthesized from dietary carbohydrates [57].

The inclusion of 10% LPRP showed similar performance to the control group and even resulted in slight improvements in protein content of larvae, indicating that partial substitution of conventional feed ingredients is feasible. Although the environmental benefits of such substitution at low inclusion levels may appear limited at the experimental scale, the valorization of underutilized peatland biomass could become more relevant when applied at larger production scales within circular bioeconomy systems. Moreover, partial replacement of commonly used feed ingredients such as wheat bran may help reduce competition with conventional feed resources. However, the economic feasibility of LPRP utilization remains uncertain and is likely to depend on factors such as biomass availability, processing requirements, and logistics, which should be addressed in future research.

## 5. Conclusions

Yellow mealworm larvae can be successfully reared on diets containing organic by-products mixed with LPRP, demonstrating the potential of these alternative ingredients in sustainable insect production systems. However, the results indicate that the LPRP inclusion level is a key determinant of larval performance and nutritional quality. Moderate inclusion (10% LPRP) showed similar performance to the control group while resulting in numerically higher protein content compared with the other groups, indicating a suitable balance between nutrient availability and dietary composition, whereas higher inclusion levels may impair nutrient utilization, likely due to increased crude fiber content and reduced digestibility.

Overall, these findings highlight that insect feed formulation should not focus solely on crude protein content, but must also consider nutrient balance, amino acid availability, and digestibility to ensure optimal growth and nutritional outcomes. This study supports the combination of LPRP with agro-industrial by-products for mealworm production within a circular economy framework while emphasizing the need for careful diet optimization. Further research is required to refine inclusion levels of LPRP and other by-products to maximize both production efficiency and nutritional quality of TM larvae for feed and food applications.

## Figures and Tables

**Table 1:** Analyzed chemical composition of lignocellulose-rich organic products and agro-industrial byproducts used in the different diets.

Item, % Dry Matter Basis	LPRP	DDGS	WB
Dry matter	87.5	90.0	89.3
Crude ash	3.39	4.74	6.14
Crude protein	9.50	32.9	17.7
Crude fiber	37.1	10.3	11.2
Crude fat	1.46	7.94	5.11

LPRP: Lignocellulose-rich organic products from rewetted peatlands; DDGS: Distillers’ dried grains with solubles; WB: Wheat bran.

**Table 2:** Diet composition and chemical composition of control and experimental diets.

Diet Composition (g/100 g Diet)	CON	LPRP			
		10%	20%	30%	40%
LPRP	0.00	10.0	20.0	30.0	40.0
DDGS	21.5	26.9	32.3	37.7	43.1
Wheat bran	78.5	63.1	47.7	32.3	16.9
Chemical analysis (% dry matter)					
Crude ash	5.68	5.46	5.07	4.76	4.53
Crude protein	21.2	20.9	21.0	20.8	21.3
Crude fiber	10.2	12.8	14.7	17.7	19.1
Crude fat	5.34	5.34	5.15	4.75	4.41
Calculated NfE	51.7	50.1	48.1	45.6	45.0

CON: Control diet; LPRP: Lignocellulose-rich organic products from rewetted peatlands; DDGS: Distillers’ dried grains with solubles; NfE: Nitrogen-free extract.

**Table 3:** Calculated amino acid content (g/100 g of protein) of the diets used for *Tenebrio molitor* larvae feeding.

Item	CON	LPRP			
		10%	20%	30%	40%
Arginine	6.92	6.28	5.64	5.00	4.35
Histidine	2.86	2.63	2.41	2.18	1.95
Isoleucine	3.41	3.42	3.43	3.44	3.45
Leucine	7.02	7.06	7.10	7.15	7.19
Lysine	4.17	3.98	3.79	3.60	3.41
Methionine	1.52	1.53	1.55	1.56	1.58
Phenylalanine	4.19	4.23	4.28	4.32	4.37
Threonine	3.58	3.56	3.53	3.51	3.48
Valine	5.32	5.24	5.16	5.09	5.01
Alanine	5.25	5.16	5.07	4.99	4.90
Aspartic acid	7.74	7.53	7.32	7.10	6.89
Cysteine	2.00	1.92	1.84	1.77	1.69
Glycine	5.71	5.45	5.19	4.93	4.67
Glutamic acid	23.8	22.90	21.90	21.00	20.10
Proline	6.70	6.69	6.68	6.68	6.67
Serine	4.60	4.53	4.46	4.38	4.31
Tyrosine	2.30	2.27	2.24	2.21	2.18
Essential amino acids	38.90	37.90	36.80	35.80	34.70
Total amino acids	97.10	94.30	91.60	88.90	86.20

CON: Control diet; LPRP: Lignocellulose-rich organic products from rewetted peatlands.

**Table 4:** Dry matter content and chemical composition (% on dry matter basis) of 10-week-old *Tenebrio molitor* larvae grown on experimental diets (mean ± standard deviation; *n* = 2).

Item	CON	LPRP			
		10%	20%	30%	40%
Dry matter	36.70 ± 0.35	36.20 ± 0.14	36.30 ± 0.42	30.60 ± 2.33	33.80 ± 2.82
Crude ash	3.98 ± 0.07	4.07 ± 0.04	4.09 ± 0.16	4.00 ± 0.18	4.12 ± 0.31
Crude protein	54.80 ± 0.80	55.80 ± 0.31	55.30 ± 1.00	53.60 ± 1.25	52.10 ± 0.72
Crude fat	32.90 ± 1.10	32.40 ± 0.92	33.00 ± 0.94	32.00 ± 0.01	33.20 ± 0.26

CON: Control diet; LPRP: Lignocellulose-rich organic products from rewetted peatlands.

**Table 5:** Levels of amino acids of *Tenebrio molitor* larvae expressed as a percentage of crude protein (mean ± standard deviation; *n* = 2).

Item	CON	LPRP			
		10%	20%	30%	40%
Arginine	5.45 ± 0.04	5.33 ± 0.58	5.74 ± 0.16	5.58 ± 0.42	4.52 ± 0.07
Histidine	2.95 ± 0.09	2.88 ± 0.33	3.02 ± 0.08	3.16 ± 0.02	2.75 ± 0.07
Isoleucine	4.28 ± 0.11	4.27 ± 0.24	4.45 ± 0.15	4.68 ± 0.09	4.30 ± 0.00
Leucine	6.72 ± 0.06	6.91 ± 0.26	6.81 ± 0.28	7.35 ± 0.30	6.79 ± 0.05
Lysine	5.43 ± 0.08	5.24 ± 0.58	5.53 ± 0.22	5.77 ± 0.07	5.27 ± 0.00
Methionine	1.34 ± 0.00	1.18 ± 0.08	1.20 ± 0.05	1.28 ± 0.05	1.26 ± 0.09
Phenylalanine	3.52 ± 0.03	3.47 ± 0.37	3.69 ± 0.12	3.96 ± 0.07	3.50 ± 0.03
Threonine	3.57 ± 0.32	3.32 ± 0.40	4.01 ± 0.10	3.96 ± 0.26	3.53 ± 0.37
Valine	6.57 ± 0.21	6.35 ± 0.73	6.75 ± 0.31	7.42 ± 0.29	7.13 ± 0.41
Tryptophan	ND	ND	ND	ND	ND
Alanine	7.51 ± 0.26	7.26 ± 0.67	7.66 ± 0.30	8.38 ± 0.11	7.77 ± 0.07
Aspartic acid	7.62 ± 0.07	7.31 ± 0.97	7.82 ± 0.31	8.30 ± 0.17	7.56 ± 0.09
Cysteine	0.94 ± 0.06	0.76 ± 0.00	0.86 ± 0.09	0.83 ± 0.06	0.93 ± 0.01
Glycine	5.09 ± 0.11	5.01 ± 0.53	5.28 ± 0.19	5.74 ± 0.08	5.29 ± 0.06
Glutamic acid	13.6 ± 0.23	13.6 ± 1.55	14.4 ± 0.50	15.3 ± 0.39	13.7 ± 0.16
Proline	5.40 ± 0.12	5.19 ± 0.51	5.60 ± 0.22	6.02 ± 0.22	5.87 ± 0.16
Serine	4.43 ± 0.11	4.36 ± 0.42	4.60 ± 0.24	5.00 ± 0.10	4.28 ± 0.26
Tyrosine	6.04 ± 0.04	6.94 ± 0.48	6.05 ± 0.36	6.83 ± 0.03	5.67 ± 0.18
Essential amino acids	39.8	38.9	41.2	43.1	39.0
Total amino acids	90.5	89.3	93.5	99.6	90.2

CON: Control diet; LPRP: Lignocellulose-rich organic products from rewetted peatlands. ND: Not determined (tryptophan was not analyzed in this study).

## Data Availability

The data presented in this study are available in this manuscript; further inquiries can be directed to the corresponding author.

## Abbreviations

The following abbreviations are used in this manuscript:

CON	Control diet
DDGS	Distillers’ dried grains with solubles
FCR	Feed conversion ratio
LPRP	Lignocellulose-rich organic products from rewetted peatlands
NDF	Neutral detergent fiber
NfE	Nitrogen-free extract
TM	*Tenebrio molitor*
WB	Wheat bran
